# Clinical Studies on the Application of Concentrated Growth Factors for Enhancing the Recovery From Flap Ischemia–Reperfusion Injuries

**DOI:** 10.1111/jocd.70203

**Published:** 2025-04-23

**Authors:** BoQiao Zhou, XianYan Luo

**Affiliations:** ^1^ Department of Dermatology Hangzhou Third People's Hospital Zhejiang Hangzhou China

**Keywords:** concentrated growth factor, flap ischemia–reperfusion injury, survival rate, wound healing

## Abstract

**Objective:**

To evaluate the therapeutic potential of locally injecting concentrated growth factor (CGF) into flaps for treating flap ischemia–reperfusion injury (FIRI) following reconstructive surgery.

**Methods:**

Nineteen patients with FIRI were enrolled in this study. After clinical identification of ischemia–reperfusion (IR) injury, CGF was prepared from each patient's autologous blood and locally injected into the affected flap tissue. Flap viability was assessed using clinical indicators such as color, texture, and capillary refill. Patients were followed for 6 months postoperatively to assess long‐term outcomes.

**Results:**

Seven days after CGF injection, 18 of 19 patients demonstrated complete flap survival. In one case, total necrosis occurred due to hematoma formation and insufficient dressing compression, but healing was achieved through dressing changes. Overall, patients showed improvements in flap color (from dark to ruddy), texture (softened), and skin tension (decreased), with visible wrinkle formation. Long‐term follow‐up confirmed satisfactory appearance and functional outcomes.

**Conclusion:**

Local injection of CGF effectively enhances flap survival by promoting recovery from IR injury. This strategy may offer a promising adjunctive therapy in reconstructive procedures to improve tissue viability and healing.

## 1 Introduction

Flap ischemia–reperfusion injury (FIRI) refers to the exacerbation of damage to a flap following prolonged ischemia and subsequent restoration of blood flow or oxygen supply [1]. Flap transplantation is a surgical technique in microsurgery that is utilized to treat severe tissue defects resulting from trauma, infection, or tumor excision surgery. It is commonly used in clinical practice to treat skin defects in areas such as the head, face, trunk, and limbs [2]. Previous research has demonstrated that the success rate of free flap transplantation is approximately 90%. However, about 10% of patients may experience flap necrosis postoperatively due to complications [3]. Therefore, it is important to address the ischemia–reperfusion (IR) injury that occurs after flap transplantation, as it is a major contributing factor to free flap necrosis. Ensuring a good blood supply to the flap is key to the survival of various types of flaps during the process of transferring the flap to repair missing skin.

Concentrated growth factor (CGF) is a plasma derived from centrifuged whole blood that is rich in platelets and contains a high concentration of growth factors, including vascular endothelial growth factor (VEGF), epidermal growth factor (EGF), and basic fibroblast growth factor (bFGF), among others [4]. These growth factors can enhance vascular permeability and expedite the migration of endothelial cells, thereby facilitating the formation of new blood vessels. CGF is commonly used in treating skin wounds, including the restoration of atrophic scars. The timing of blood supply and the quality of regenerated blood vessels in adipose tissue transplanted to the recipient area are crucial for the ultimate restoration of atrophic scars. CGF contains a significant quantity of transforming growth factor beta 1 (TGF‐β1), VEGF, and other growth factors, along with CD34+ cells and fibrin. These components can bind to receptors on target cells, transmitting signals that regulate the metabolic activity of the cells. This promotes angiogenesis and vascular maintenance. Kao conducted a treatment trial with CGF on 18 patients with venous ulcers [5]. The study demonstrated that CGF gel accelerated the regeneration of soft tissue at the wound site, while CGF membrane promoted re‐epithelialization at its edges. The combination of both treatments effectively and safely promoted autologous regeneration of deep and superficial wounds. In vitro experiments have shown that CGF promotes the migration and proliferation of HaCaT cells, leading to the formation of epithelial‐like structures [6]. This suggests that CGF can help with soft tissue regeneration and re‐epithelialization at wound edges. Studies, such as those by Kabilamurthi et al. have indicated that CGF can accelerate wound healing and improve the quality of wound healing after implant surgery in cases of acute wound injuries [7]. Calabriso et al. found that besides growth factors, CGF also contains a significant amount of MMP‐2 and MMP‐9. MMP‐9 can selectively degrade the extracellular matrix, recruit endothelial cells, and assist endothelial cell migration to budding sites, thereby promoting vascular regeneration [8].

CGF comprises autologous cells, leukocytes, platelet‐derived growth factors (PDGFs), and a fibrin scaffold, which collectively create a biomimetic environment for tissue regeneration and healing. Furthermore, CGF can promote the recruitment and proliferation of specific cells, inducing regeneration of the damaged wound surface. Therefore, it plays a significant role in acute and chronic wound surfaces, as well as complex infected wound surfaces. It is hypothesized that concentrated cell factors may have a similar effect on flap ischemia–reperfusion injuries, thereby improving flap survival rates. This paper presents clinical research investigating a scientific question [9].

## 2 Patients and Methods

### 2.1 Clinical Data

Between May 2022 and October 2023, 19 patients suffering from flap ischemia–reperfusion injuries were treated with CGF at the Department of Dermatological Surgery in The Third People's Hospital of Hangzhou City. The cohort included 8 males and 11 females, ranging in age from 51 to 86 years. These patients underwent radical excision of skin lesions, followed by pedicled flap transplantation and cosmetic suturing. They experienced IR injuries during their first postoperative dressing change, after which they consented to local injections of CGF. All examinations and procedures were conducted following the guidelines of the Declaration of Helsinki, and informed consent was obtained from all patients before treatment, with approval from the hospital's ethics committee.

The criteria for patient selection were based on several factors. Firstly, the age range was set at 50 to 90 years to ensure the study included a middle‐aged to elderly population. Secondly, the inclusion criteria included patients who showed moderate to severe IR injuries following flap transplantation. These injuries typically manifest as changes in flap coloration, increased local pain, or restricted flap functionality. Additionally, overall health conditions were considered, excluding patients with severe cardiovascular, renal, or hepatic diseases to minimize the risk of postoperative complications.

By implementing these stringent selection criteria, the study aimed to ensure that the included patient population could safely undergo treatment while also securing the reliability and validity of the data. Specific patient data and treatment details are presented in Table 1.

**TABLE 1:** Clinical characteristics and treatment of 19 patients treated with CGF.

No./gender/age	Flap location	Flap area (cm × cm)	Cause of injury	No. of injections	FIRI onsite (h)	Underlying conditions	Flap survival status	Flap recovery area (%)
1/F/76	Left facial	4.5 × 2.3	BCC	1	18	None	Complete recovery	100
2/M/65	Right facial	3.1 × 2.2	BCC	2	20	Hypertension	Good recovery	75
3/M/76	Left medial canthus	3.9 × 1.5	BCC	1	24	Hypertension	Moderate recovery	50
4/F/84	Right nasolabial fold	2.2 × 1.6	BCC	2	24	Hypertension, atrial fibrillation	Complete recovery	100
5/M/51	Left lower eyelid	4.2 × 1.6	BCC	1	24	None	Good recovery	75
6/F/75	Left temporal	5.6 × 1.5	BCC	2	24	None	Complete recovery	100
7/F/86	Left cheek	6.1 × 1.3	BCC	2	24	Coronary artery disease	Complete recovery	100
8/F/57	Left temporal	3.9 × 1.5	BCC	2	24	Type 2 diabetes, hepatitis B	Good recovery	75
9/M/81	Right lower eyelid	2.9 × 1.5	BCC	1	24	None	Complete recovery	100
10/F/85	Left beside nose	2.1 × 1.5	BCC	2	24	Hepatitis B	Complete recovery	100
11/M/71	Left facial	3.9 × 1.8	BCC	2	24	Hypertension	Moderate recovery	50
12/F/61	Right facial	1.9 × 0.8	BCC	1	24	Type 2 diabetes	Complete recovery	100
13/F/83	Right facial	3.9 × 2.5	SCC	1	24	None	Complete recovery	100
14/M/68	Left facial	4.9 × 2.1	BCC	1	24	None	Good recovery	75
15/M/71	Right ala of nose	2.3 × 2.5	BCC	1	24	Atrial fibrillation, hypertension	No recovery	0
16/M/86	Right facial	2.0 × 1.5	BCC	1	24	Hypertension, coronary artery disease	Good recovery	75
17/F/72	Left lower eyelid	3.9 × 0.5	BCC	1	24	None	Complete recovery	100
18/F/56	Right eyebrow	3.5 × 1.6	BCC	1	24	None	Good recovery	75
19/F/78	Left upper eyelid	2.9 × 1.1	BCC	2	24	None	Good recovery	75

### 2.2 Model Evaluation

The evaluation process of flap ischemia–reperfusion injuries, specifically in the context of diagnosis, assessment, and quantification before and after the injection of CGF, is a critical aspect in reconstructive surgery.

FIRI is diagnosed based on a combination of clinical assessment, imaging studies, and biochemical markers. Clinically, the flap is observed for color, capillary refill, temperature, turgor, and the presence of edema. These parameters help assess the viability of the flap and the extent of ischemic damage. Laser Doppler imaging (LDI) and indocyanine green angiography (ICGA) are commonly used to assess blood flow and perfusion in the flap. These imaging modalities provide real‐time, quantitative data on perfusion dynamics. Thermography can be utilized to detect changes in the surface temperature of the flap, which is indicative of underlying perfusion changes. Measurement of systemic and local biomarkers, such as lactate, creatine kinase, and myoglobin, is important. Elevated levels suggest tissue damage due to IR. Pro‐inflammatory cytokines (e.g., TNF‐α, IL‐6) can also be monitored as they are typically elevated during reperfusion injury.

The severity of ischemic injury is graded based on the extent of tissue necrosis, perfusion deficits, and biochemical marker levels. The flap viability index or perfusion scores derived from imaging results are used to quantify the degree of ischemia. These indices are pivotal in determining the immediate therapeutic approaches and long‐term outcomes.

### 2.3 Isolation of CGF

**FIGURE 1:**
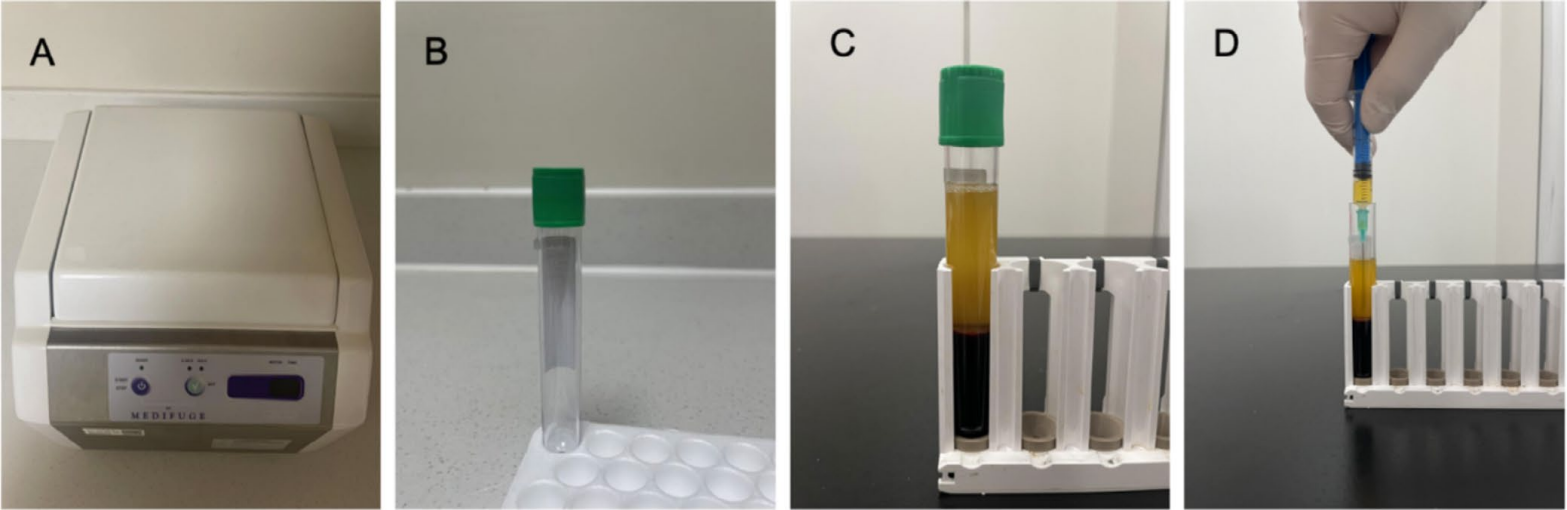
Schematic representation of the CGF preparation process. (A) The variable speed centrifuge produced by Silfradent Srl, Italy. (B) The specialized vacuum test tubes used for venous blood collection. (C) The blood shows separation into layers after centrifugation. (D) The extraction of liquid CGF using a 2.5 mL syringe.

CGF was derived from the patients' own blood through separation. Venous blood is collected from patients using 9 mL specialized vacuum test tubes, commonly referred to as “green tubes” (Product #455051; Silfradent Srl). This specific type of tube is designed to enhance the separation of blood components during centrifugation. The green tubes are immediately placed into a variable speed centrifuge (Model #MF200; Silfradent Srl). The centrifugation parameters are crucial for optimal separation. The tubes are centrifuged at a specific speed of 1500–2000 rpm. The duration of centrifugation is set at 8 min, separating the whole blood into distinct layers without causing disruption to the cellular components. Post‐centrifugation, the tubes are carefully removed to avoid mixing of the layers. Approximately 2 mL of CGF is carefully extracted using a 2.5 mL sterile syringe to prevent contamination and ensure the integrity of the CGF. This extraction is performed under aseptic conditions to maintain sterility. The CGF is then stored at a constant temperature of 15°C in a refrigerated environment to preserve its viability and functional properties. To ensure the purity and quality of the CGF, the entire procedure is conducted under strict aseptic conditions to prevent microbial contamination and cross‐infection. Regular calibration of the centrifuge and meticulous maintenance of refrigeration units are performed to ensure consistent and reliable results. The enhanced process of CGF preparation is illustrated in Figure 1, which details each step from blood collection to CGF extraction and storage. The figure includes annotations for centrifugation settings, layer identification, and handling precautions to aid in the replication of the process.

### 2.4 Injection of CGF

The patient is positioned supine, and the wound area around the ischemic flap is disinfected and draped using sterile techniques. A 1 mL syringe equipped with a needle ranging in length from 0.5 to 1.5 cm is prepared for the injection of CGF. The needle size is based on the thickness of the flap and the required depth at which the CGF needs to be delivered to effectively target the ischemic zones.

The needle is inserted at a depth appropriate for the specific flap tissue, generally between 0.3 and 1.0 cm beneath the skin surface, to ensure that the CGF is delivered directly into the subdermal plexus where it can be most beneficial. Typically, the volume of CGF injected ranges from 0.1 to 0.5 mL per injection site, depending on the size and extent of the ischemic area. The total volume administered should not exceed the amount of CGF available from extraction, which is about 2 mL per processing cycle.

The syringe is inserted at various angles to create a fan‐like pattern beneath the flap. This technique ensures broader coverage and more uniform distribution of CGF throughout the ischemic tissue. The CGF is injected slowly as the needle is gradually retracted. This method helps in dispersing the CGF along the needle track, maximizing tissue exposure to the growth factors. After the injection, a strip of Vaseline gauze is placed over the wound to minimize exposure to air and potential contaminants. The area is then covered with a standard dressing to secure the gauze in place and support the healing environment. Postoperative care involves monitoring the flap during the first dressing change to evaluate the ischemic condition and assess the initial response to the CGF treatment. Adjustments to subsequent CGF injections may be necessary based on the observed efficacy and any changes in the tissue's ischemic status.

### 2.5 Postoperative Treatment

Postoperatively, the patient is administered appropriate antibiotics and anti‐inflammatory drugs. An evaluation of the flap and its surrounding area is performed before and after each treatment session. The evaluation documents the patient's self‐reported observations regarding the wound, treatment duration, and healing progress.

### 2.6 Potential Limitations

This study explored the potential therapeutic effects of local injections of CGF for the recovery from FIRI. However, several limitations must be considered when interpreting the findings. Although most patients responded positively to CGF injections, some flaps did not survive. This variability may be associated with factors such as the patients' underlying health conditions, the original state of the flap, and specific surgical techniques used. Despite efforts to control for confounding factors through selection criteria and statistical analysis, there may still be variables that were not accounted for, such as patients' lifestyles, medication histories, and other unrecorded medical conditions, which could affect the outcomes of CGF treatment.

Meantime, the study primarily used observations of changes in flap color, texture, and capillary refill tests to evaluate treatment efficacy. While practical, these methods may lack sufficient objectivity and reproducibility. Future studies could consider incorporating more objective biomarkers or imaging techniques to enhance the accuracy and reliability of assessments. The small sample size (19 patients) was limited to patients treated at a single hospital. Therefore, the results may not be generalizable to a broader population.

## 3 Result

The survival of the flap is assessed 7 days after the initial injection based on its appearance, texture, and the capillary refill test. The boundary between necrotic and viable areas of the flap is marked, and photographs are taken for documentation purposes. The survival status of the flap is recorded, and the survival rate is calculated accordingly. As shown in Table 1, the color of the flap changed from dark to ruddy after CGF injection for 19 patients. Additionally, some parts of the flap softened in texture, skin wrinkles appeared, tension decreased, and the capillary refill time was shorter compared to before the treatment. However, in one case, the flap completely necrosed due to loosening at the injection site. This resulted in ineffective compression by the bandage and the accumulation of blood, which adversely affected the healing of the flap. The survival rate of the flap 7 days after injection was 80.3% ± 26.5%.

Clinically, 50% of the patients showed improvement in the damaged flap after the first CGF injection. This was indicated by partial discoloration from dark to ruddy on the skin. The condition of the wound surface became stable 7 days post‐CGF injection, with the incision surface gradually healing. The post‐surgery follow‐up revealed that the flaps that responded well to the injection remained viable and integrated with the normal tissue, with no significant scarring. Additionally, the color of the flap was similar to the surrounding healthy skin, which contributed to increased patient satisfaction.

### 3.1 Case 1

**FIGURE 2:**
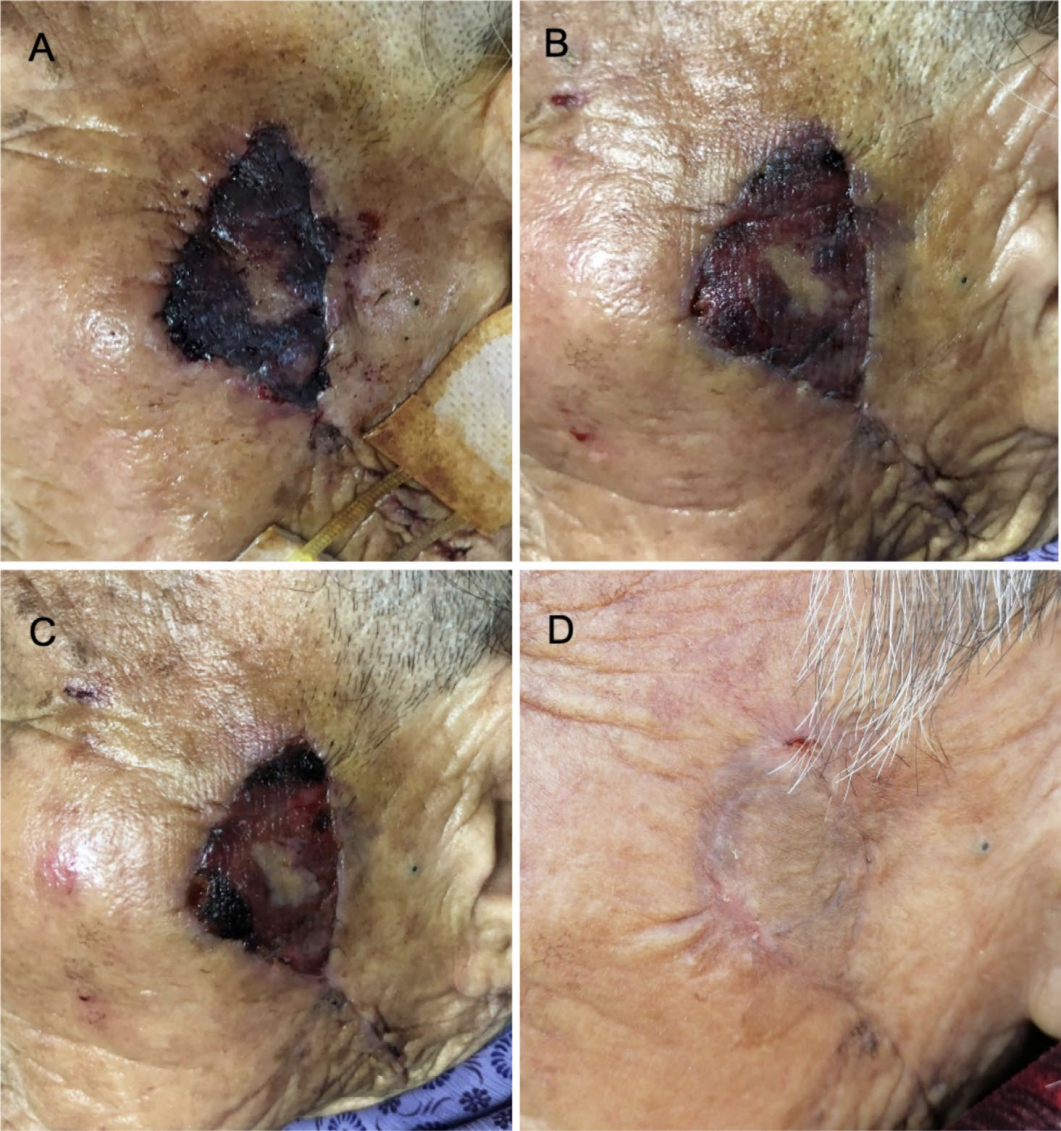
The effects of CGF treatment on an elderly female patient who experienced FIRI postoperatively. (A) FIRI and partial flap necrosis occurred when the wound was dressed on the first day after surgery. On the same day, the patient's own venous blood was drawn, centrifuged to prepare CGF, and the first CGF injection treatment was administered. (B) Seventy‐two hours after the first CGF injection, the condition of the wound showed improvement, with the flap color changing from bruised to red, and swelling reduced compared to before. (C) Seven days after the first CGF injection, the flap appeared healthy and red, swelling had subsided, and the skin survival rate reached 75%. (D) Forty‐two days after the first CGF injection, the flap had completely healed.

The 76‐year‐old female patient had a skin lesion on the left side of her face that was treated by radical excision, pedicled flap transplantation, and cosmetic suturing. The flap was discovered to be bruised, purple, slightly swollen, under increased stress, and to have a shortened capillary response during the wound dressing change 18 h after the surgery. The black blood flowing slowly after the needle puncture implies that the flap was injured by ischemia. The patient had her initial injection of CGF. On the second day following the injection, the edema had subsided, the color of the wound flap had changed to red, and a needle puncture revealed fresh blood. Every other day, the dressing was changed After the injection, the flap had healed properly within 7 days. As shown in Figure 2, the incision healed fully after 42 days with no hypertrophic scar or subcutaneous induration.

## 4 Discussion

Flap transfer, a cornerstone procedure in the field of plastic and reconstructive surgery, varies significantly among different flap types. The fundamental prerequisite for the success of any flap transfer is the establishment of an optimal blood supply, which is essential for the survival and integration of the transferred tissue. CGF, a promising therapeutic adjunct, is a platelet‐rich fibrin matrix that encompasses a constellation of growth factors pivotal to tissue repair and regeneration. These include PDGF, EGF, FGF, TGF‐β, and VEGF [10]. Insulin‐like growth factor‐1 (IGF‐1), a key component of CGF, has been shown to stimulate the migration and proliferation of endothelial cells, thereby facilitating the formation of vascular channels. This process is instrumental in the development of a robust neovascular network [11]. Furthermore, IGF‐1 promotes the migration of vascular smooth muscle cells and perivascular cells, which are crucial for the structural integrity and stability of newly formed blood vessels. Collectively, these actions lead to enhanced angiogenesis [12], an increase in the number of blood vessels, and the establishment of a more extensive and voluminous vascular network. This, in turn, expands the reach and capacity of blood supply, mitigating ischemic and hypoxic damage to the flap and fostering its survival. The biological activity of CGF is largely attributed to its natural fibrin scaffold, which provides a biocompatible and biodegradable matrix for cell adhesion, proliferation, and differentiation. The fibrin network degrades autonomously, releasing a cascade of growth factors and platelets that further augment the healing process. This dynamic release mechanism ensures a sustained supply of bioactive molecules, optimizing the wound healing environment. Mesenchymal stem cells (MSCs), which can be found within the CGF matrix, exhibit a synergistic relationship with the various endogenous growth factors. They contribute to angiogenesis and tissue reconstruction, hastening the wound repair process. The paracrine effects of MSCs, along with the growth factors, create a conducive microenvironment that promotes the proliferation and migration of epithelial cells, thereby facilitating the growth and regeneration of various epithelial tissues. In conclusion, the multifaceted therapeutic potential of CGF, stemming from its rich growth factor profile, natural fibrin scaffold, and the synergistic action of MSCs, positions it as a valuable asset in the armamentarium of reconstructive surgery. Its ability to modulate the wound healing cascade, promote angiogenesis, and enhance tissue regeneration underscores its significance in optimizing flap survival and overall surgical outcomes. Future research should aim to elucidate the precise mechanisms of action of CGF and explore its potential synergies with other therapeutic modalities to further refine flap transfer techniques and improve patient outcomes [13].

CGF has been widely used in clinical settings due to its significant safety advantages. As CGF is derived from the patient's own blood, it eliminates the risk of infectious diseases and avoids immune rejection, enhancing the safety of its clinical application. It has been extensively used in clinical dentistry [14], maxillofacial bone repair [15], and plastic surgery [16], showing considerable potential in promoting the repair and regeneration of other tissues. In the context of tissue injury repair, some studies have identified that CGF releases growth factors within 24 h of application, providing a high concentration of growth factors with sustained release, thereby facilitating cell proliferation and tissue differentiation [17, 18]. In vitro studies suggest that CGF can significantly enhance fibroblast proliferation in the healing of skin wounds [19]. Based on the mechanisms by which CGF promotes vascular regeneration and our research findings, local CGF injections have been shown to encourage neovascularization, reduce inflammatory responses, accelerate healing, and increase flap survival rates. These results suggest that CGF has beneficial effects on flaps, providing a new clinical perspective for addressing flap ischemic injuries.

In this comprehensive study, encompassing a cohort of 19 patients, we observed a survival rate of flaps that surpassed the 80% threshold, which underscores the profound therapeutic efficacy of CGF in mitigating ischemic reperfusion injuries within flaps. The CGF enhances tissue repair and regeneration through its rich content of growth factors, cytokines, and other bioactive molecules. These components are instrumental in modulating the inflammatory phase, promoting angiogenesis, and facilitating the migration and proliferation of cells crucial for wound healing. However, our study also encountered a critical incident where one patient experienced flap necrosis secondary to a subcutaneous hematoma. This complication arose from the patient's inadvertent loosening of their wound dressing due to discomfort, leading to a disruption in the microenvironment necessary for optimal flap survival. This case underscores the critical need for innovative approaches in dressing and fixation methods to ensure the integrity of the wound site and to mitigate patient discomfort.

To address this, future research should focus on the development of biocompatible and patient‐friendly dressing materials that provide a conducive environment for healing, while minimizing discomfort and the risk of displacement. Additionally, the exploration of advanced fixation techniques that offer superior stability without compromising the flap's vascularity and the patient's comfort is of paramount importance.

## 5 Conclusion

The local color of the flap changed from dark to ruddy, the texture of the flap softened, skin wrinkles appeared, and tension decreased. This demonstrates that local injection of CGF into flaps postoperatively can effectively improve IR injury.

## Conflicts of Interest

The authors declare no conflicts of interest.

## Data Availability

The data that support the findings of this study are available from the corresponding author upon reasonable request.
